# Supplementary figures and images for: Ribosome profiling of porcine reproductive and respiratory syndrome virus reveals novel features of viral gene expression
Source: eLife. 2022 Feb 28;11:e75668. doi: 10.7554/eLife.75668 (PMC9000960; doi:10.7554/eLife.75668)

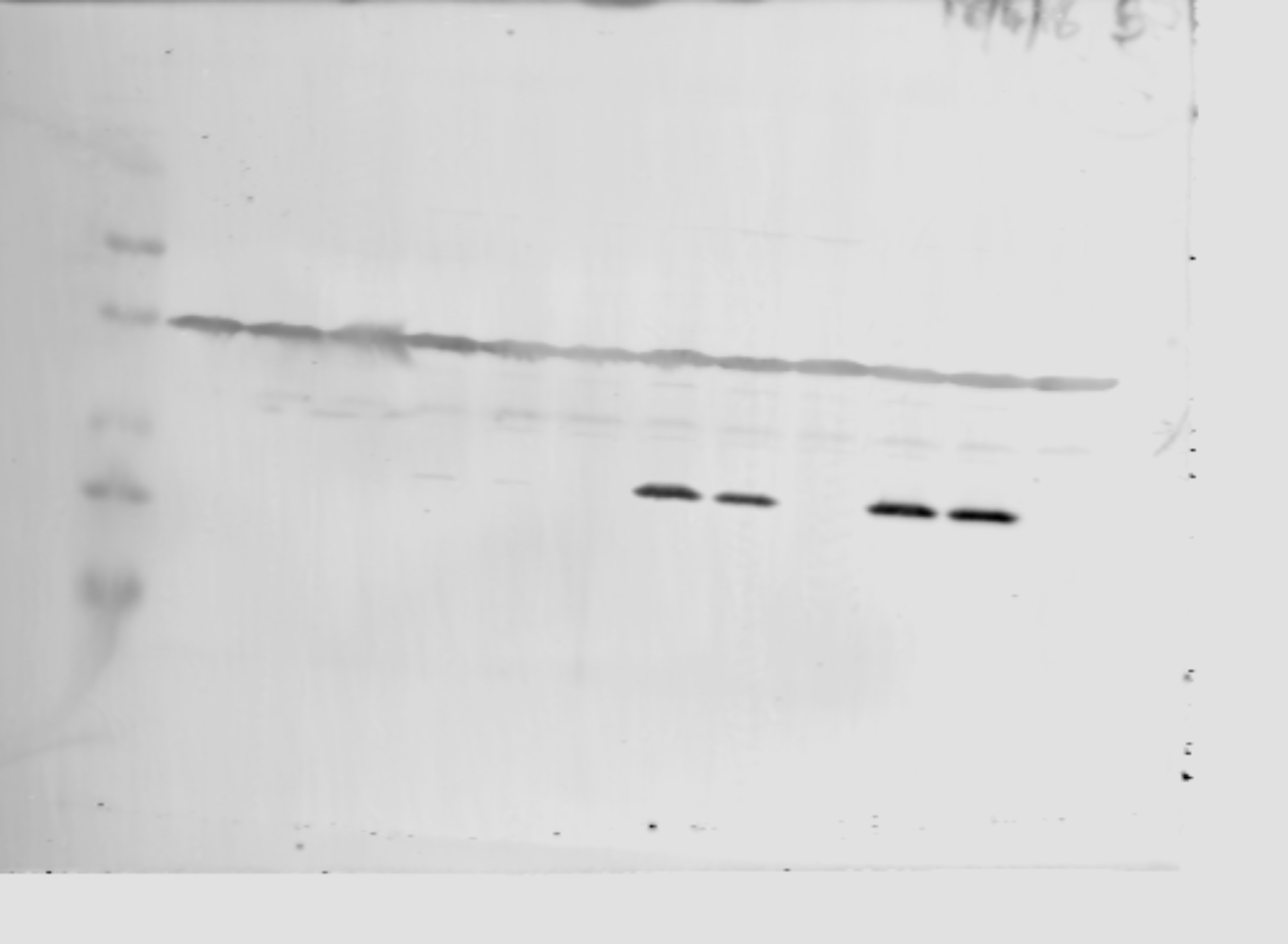

Supplement: Figure 3—source data 1. [file elife-75668-fig3-data1.tif]

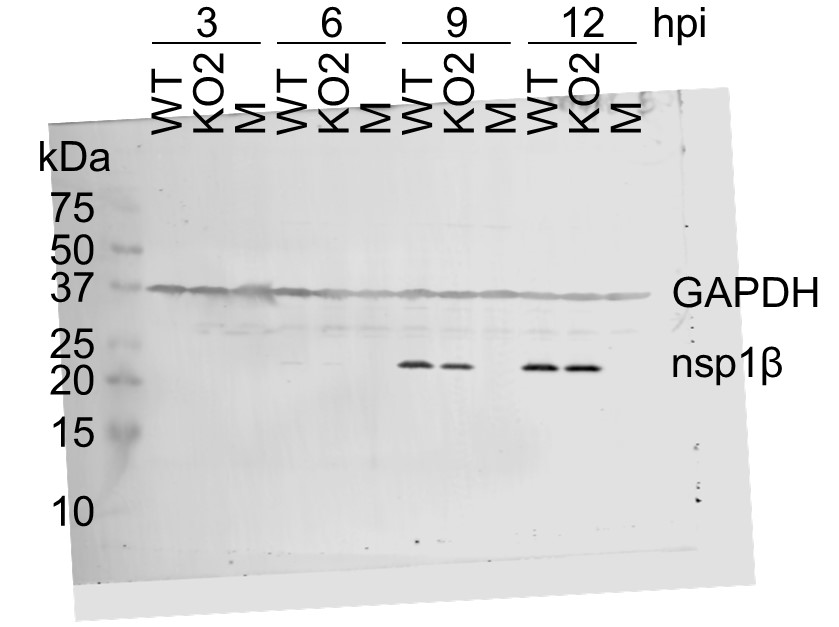

Supplement: Figure 3—source data 2. [file elife-75668-fig3-data2.jpg]

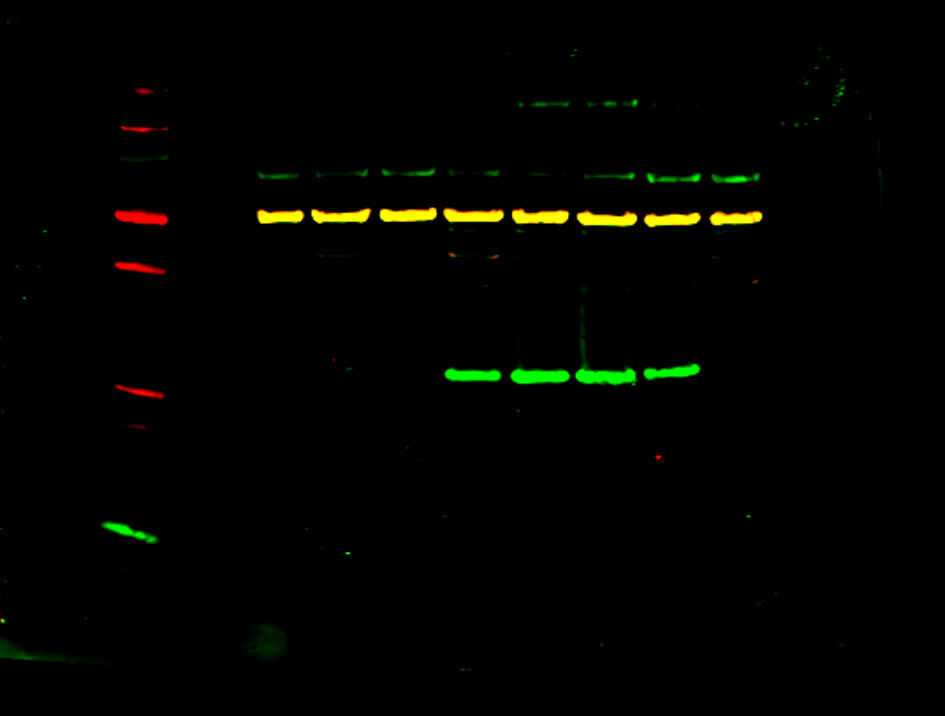

Supplement: Figure 3—source data 4. [file elife-75668-fig3-data4.tif]

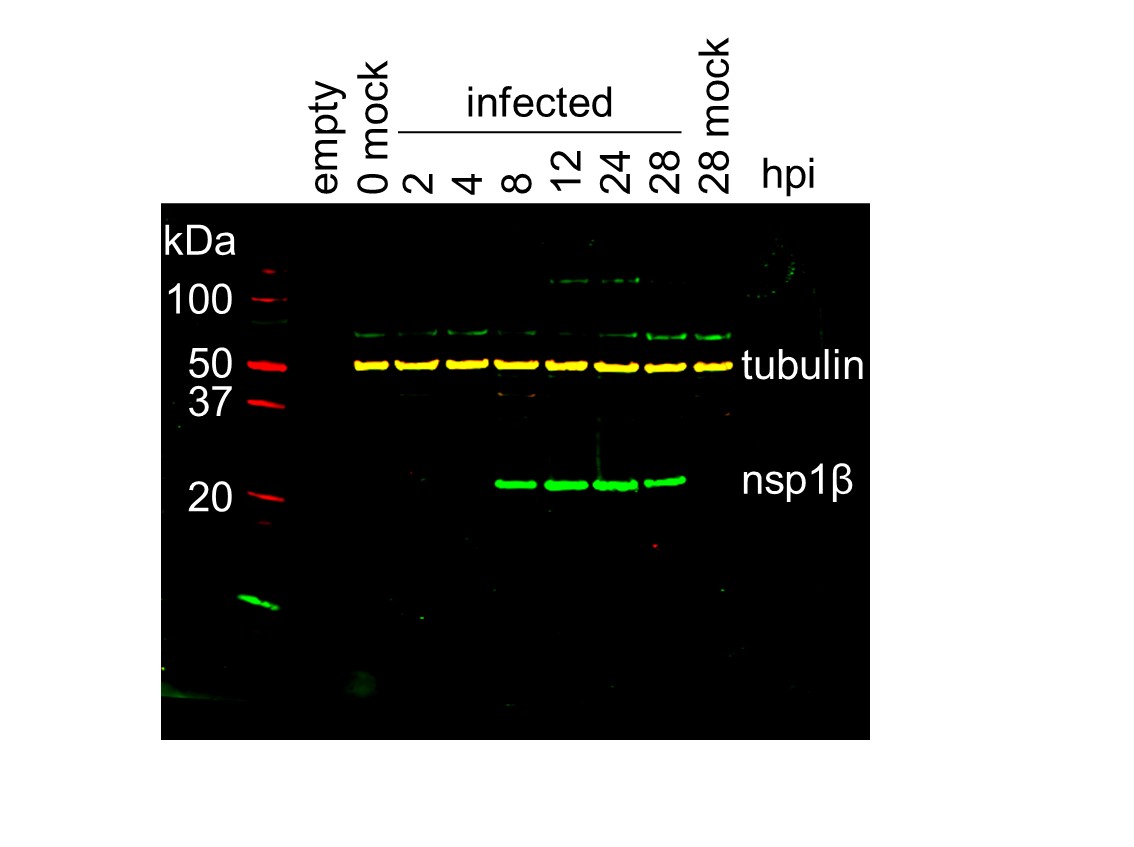

Supplement: Figure 3—source data 5. [file elife-75668-fig3-data5.jpg]
