# Supplementary material for: Ribosome profiling of porcine reproductive and respiratory syndrome virus reveals novel features of viral gene expression
Source: eLife. 2022 Feb 28;11:e75668. doi: 10.7554/eLife.75668 (PMC9000960; doi:10.7554/eLife.75668)
Supplement: Transparent reporting form [file elife-75668-transrepform1.docx]

***eLife’s* transparent reporting form**

We encourage authors to provide detailed information *within their submission* to facilitate the interpretation and replication of experiments. Authors can upload supporting documentation to indicate the use of appropriate reporting guidelines for health-related research (see [EQUATOR Network](http://www.equator-network.org/%20)), life science research (see the [BioSharing Information Resource](https://biosharing.org/)), or the [ARRIVE guidelines](http://www.plosbiology.org/article/info:doi/10.1371/journal.pbio.1000412) for reporting work involving animal research. Where applicable, authors should refer to any relevant reporting standards documents in this form.

If you have any questions, please consult our Journal Policies and/or contact us: [editorial@elifesciences.org](mailto:editorial@elifesciences.org).

**Sample-size estimation**

- You should state whether an appropriate sample size was computed when the study was being designed
- You should state the statistical method of sample size computation and any required assumptions
- If no explicit power analysis was used, you should describe how you decided what sample (replicate) size (number) to use

Please outline where this information can be found within the submission (e.g., sections or figure legends), or explain why this information doesn’t apply to your submission:

Ribosome profiling is an expensive and time-consuming procedure and is not amenable to large sample sizes. Duplicate samples is typical in the field. To check whether results were reproducible we required at least two replicates for each condition, although in many cases we effectively have at least four, because the KO2 mutant virus can be treated as equivalent to the WT virus for many analyses. This is made clear throughout the text and Figures (see next section).

**Replicates**

- You should report how often each experiment was performed
- You should include a definition of biological versus technical replication
- The data obtained should be provided and sufficient information should be provided to indicate the number of independent biological and/or technical replicates
- If you encountered any outliers, you should describe how these were handled
- Criteria for exclusion/inclusion of data should be clearly stated
- High-throughput sequence data should be uploaded before submission, with a private link for reviewers provided (these are available from both GEO and ArrayExpress)

Please outline where this information can be found within the submission (e.g., sections or figure legends), or explain why this information doesn’t apply to your submission:

All replicates presented in this manuscript are biological replicates, with one dish of cells representing a single replicate – this is stated in the 'Cells and viruses' section of the Materials and Methods.

The deep sequencing data for all libraries described in this manuscript have been uploaded to ArrayExpress under the accession numbers given in the Materials and Methods (under embargo until publication but accessible to reviewers).

For many figures, replicates are displayed individually (for example Figures 1C, 2B and 2C), making the number of replicates clear from the beginning of the Results onwards, with the same datasets analysed throughout the paper. In cases where replicates are combined to generate a figure, this is stated in the figure legend (for example Figure 11).

Where datasets were excluded or their use limited to particular analyses this is explained and justified in the first section of the Results.

**Statistical reporting**

- Statistical analysis methods should be described and justified
- Raw data should be presented in figures whenever informative to do so (typically when N per group is less than 10)
- For each experiment, you should identify the statistical tests used, exact values of N, definitions of center, methods of multiple test correction, and dispersion and precision measures (e.g., mean, median, SD, SEM, confidence intervals; and, for the major substantive results, a measure of effect size (e.g., Pearson's r, Cohen's d)
- Report exact p-values wherever possible alongside the summary statistics and 95% confidence intervals. These should be reported for all key questions and not only when the p-value is less than 0.05.

Please outline where this information can be found within the submission (e.g., sections or figure legends), or explain why this information doesn’t apply to your submission:

Statistical methods are described in the Results and Materials and Methods sections, with exact *p* values provided within the text of the Results or within the Supplementary Tables pertaining to the relevant analyses. Where figures show average values for several replicates combined, the values for each individual replicate are overlaid as a scatter plot where space permits.

(For large datasets, or papers with a very large number of statistical tests, you may upload a single table file with tests, Ns, etc., with reference to sections in the manuscript.)

**Group allocation**

- Indicate how samples were allocated into experimental groups (in the case of clinical studies, please specify allocation to treatment method); if randomization was used, please also state if restricted randomization was applied
- Indicate if masking was used during group allocation, data collection and/or data analysis

Please outline where this information can be found within the submission (e.g., sections or figure legends), or explain why this information doesn’t apply to your submission:

Groups were allocated according to the experimental conditions under which the samples were harvested (timepoint; WT/KO2/mock/EU infection; inclusion/omission of CHX pre-treatment). In cases where the mutations present in the KO2 mutant virus were not expected to affect the results of a particular analysis, the WT and KO2 samples were treated as a single group, in which case this was stated in the text.

**Additional data files (“source data”)**

- We encourage you to upload relevant additional data files, such as numerical data that are represented as a graph in a figure, or as a summary table
- Where provided, these should be in the most useful format, and they can be uploaded as “Source data” files linked to a main figure or table
- Include model definition files including the full list of parameters used
- Include code used for data analysis (e.g., R, MatLab)
- Avoid stating that data files are “available upon request”

Please indicate the figures or tables for which source data files have been provided:

For Figure 3 (panels D-F), the raw western blots and quantification values are provided in Figure 3-source data 1-5.

For Figures 4 and 5, the source data can be found in Figure 4-source data 1-3 and Figure 5-source data 1-2. The data in these tables was also used to generate Figures 7 and 8 and Figure8-figure supplements 1 and 3.

For Figure 6, the source data used to annotate the ORFs is provided in Figure 6-source data 1. The data in this table was similarly used to annotate the ORFs in Figure 6-figure supplements 1 and 2 and Figure 7. The expression data in this table was used to generate some of the plots in Figure 8 and Figure 8-figure supplements 2 and 3.

For Figure 11, the source data can be found in Figure 11-source data 1-3.
